# Non-Bulk-Like Solvent Behavior in the Ribosome Exit Tunnel

**DOI:** 10.1371/journal.pcbi.1000963

**Published:** 2010-10-21

**Authors:** Del Lucent, Christopher D. Snow, Colin Echeverría Aitken, Vijay S. Pande

**Affiliations:** 1 Biophysics Program, Stanford University, Stanford, California, United States of America; 2 Department of Chemistry, Stanford University, Stanford, California, United States of America; 3 Department of Structural Biology, Stanford University, Stanford, California, United States of America; University of Houston, United States of America

## Abstract

As nascent proteins are synthesized by the ribosome, they depart via an exit tunnel running through the center of the large subunit. The exit tunnel likely plays an important part in various aspects of translation. Although water plays a key role in many bio-molecular processes, the nature of water confined to the exit tunnel has remained unknown. Furthermore, solvent in biological cavities has traditionally been characterized as either a continuous dielectric fluid, or a discrete tightly bound molecule. Using atomistic molecular dynamics simulations, we predict that the thermodynamic and kinetic properties of water confined within the ribosome exit tunnel are quite different from this simple two-state model. We find that the tunnel creates a complex microenvironment for the solvent resulting in perturbed rotational dynamics and heterogenous dielectric behavior. This gives rise to a very rugged solvation landscape and significantly retarded solvent diffusion. We discuss how this non-bulk-like solvent is likely to affect important biophysical processes such as sequence dependent stalling, co-translational folding, and antibiotic binding. We conclude with a discussion of the general applicability of these results to other biological cavities.

## Author Summary

The physical properties of water are of critical importance to biomolecular processes such as protein folding and protein-ligand binding. The predominant view of water from a structural biological perspective, however, is that of a two-state model: water is either a tightly bound molecule (as seen in x-ray crystallography) or a continuous bulk fluid of constant dielectric. When water is confined on the nanometer length scale (as it is in a number of critically important biological cavities) the two-state characterization is likely to be inadequate. Using molecular dynamics simulations we report on the thermodynamic and kinetic properties of water confined to the ribosome exit tunnel. We show that this solvent is slow-diffusing and semi-structured, sampling a great variety of states between its characteristic ‘end-points’ of bulk-like and tightly bound. We conclude with a discussion of how the properties of this nano-confined solvent can affect the interpretation of recent experimental results regarding sequence dependant stalling and co-translational folding in the ribosome exit tunnel.

## Introduction

The first microenvironment a protein experiences is that of the ribosome exit tunnel, the long cavity in the large ribosomal subunit through which the nascent peptide emerges during translation. Cryo-electron microscopy and x-ray crystallography have revealed this cavity to be about 100Å long with a diameter of 10–20 Å [1], [2]. To a certain degree, the exit tunnel must be promiscuous in its interactions, so as to facilitate the translation of disparate protein sequences [2], [3].

However, a number of protein sequences have been shown to stall translation, presumably via specific interactions with the exit tunnel. Most notable among these are the tnaC and SecM sequences [4]. Although the exact molecular mechanism for stalling has not yet been deduced, it is strongly believed that these sequences initiate stalling via interactions with the ribosome in a region where ribosomal proteins L22 and L4 protrude into the exit tunnel [4]–[6]. Lu et al have shown by site-specific chemical modification of particular nascent peptide sequences that the electrostatic potential in the tunnel is predominantly negative but also quite heterogeneous [7]. Nascent peptide sequences with stretches of positive amino acids have been shown to transiently stall translation in accordance with the known electrostatic characterization of the tunnel [8]. It has also been suggested that nascent peptides can fold to a limited extent in the tunnel [9]–[11]. For example, the SecM sequence is compacted while in the exit tunnel, and this compaction is necessary for stalling [12]. Additionally, recent cryo-electron microscopy has shown a nascent peptide folding into an alpha helix near the exit of the tunnel (a region known as the vestibule) [13]. Not all sequences may be able to fold in the tunnel however. The same group has also used cryo-EM to show that the nascent chain for the tnaC sequence adopts an expanded (non-helical) conformation in the exit tunnel [14]. In addition to these stalling sequences, there are a number of sequences that do not cause translational arrest, but have widely disparate rates of translation [8], [15], [16].

Macrolide antibiotics have been shown to bind to regions of the exit tunnel between the peptidyl transferase site and the constriction site [17], [18]. Various macrolide antibiotics, which are known to bind in the ribosome exit tunnel, can have greatly different binding modes and disparate effects on translation [19]. Mutations to the rRNA or proteins in the area of the constriction site can also have an effect on antibiotic binding, with some mutations conferring resistance to antibiotic treatment [20], [21].

The ribosome exit tunnel is a peculiar microenvironment from a nanoscopic point of view in that it is both heterogeneous (containing both polar and non-polar residues) and highly confined. Both of these characteristics are exacerbated when considering the presence of nascent peptide (which due to the natural variation of protein sequences creates an environment that is even more heterogeneous in terms of hydrophobicity and even more spatially confined). We believe that an important step in understanding the complexity of molecular behavior in the ribosome exit tunnel requires an atomistic understanding of the thermodynamics and kinetics of the residues in the tunnel as well as the solvent confined to the exit tunnel.

The behavior of water on short length scales has been studied both experimentally and via computer simulation [22]–[29]. The peculiar microenvironment created by highly confined water has been shown to have an effect on protein folding and stability [30], [31]. Additionally, water is known to organize differently in the vicinity of hydrophilic and hydrophobic surfaces [32]–[35] further complicating the potential effect on the dynamics of confined peptides. For example, it has been recently shown that the different modes of organization confined water exhibits inside chaperonins can partially explain their ability to fold and unfold proteins [36], [37]. Just as unstructured loops of proteins have large effects on their stability and activity, so may semi-structured confined water affect the behavior of nascent peptides and small molecules in the ribosome exit tunnel.

Common structural methods usually treat biologically confined water as either a discrete tightly bound molecule, or a continuous, bulk-like dielectric fluid. Based on the known structural properties of the ribosome, we hypothesize that the majority of the water found in the ribosome exit tunnel falls between these endpoints. Although nano-confined water has been studied experimentally before, none of these methods are particularly well suited to deal with the large and complex ribosome and still capture the subtleties of the solvent confined within. For this reason, molecular simulation is an ideal way to probe the solvent confined in the exit tunnel. However, due to the complexity and computational demands of simulating macromolecular complexes of the scale of the ribosome, previous attempts to simulate the ribosome have often used simplified models for the solvent or only simulated a few trajectories at relatively short timescales [38]–[43]. Here we present the results of extensive atomistic molecular dynamics simulations, performed with the intent of studying solvent confined to the ribosome exit tunnel.

## Results

We modeled the ribosome exit tunnel in all-atom detail for the ribosome and solvent. Our simulations consisted of 91,787 atoms total, including solvent, ions, and a portion of the ribosome including 85 Å surrounding the ribosome tunnel. Since a high-resolution structure showing the conformation of a nascent peptide does not yet exist, we felt it best to consider the case of an empty tunnel. This allows us to more directly probe the general nature of the exit tunnel environment in terms of the nature of the water inside of the tunnel. Moreover, it is reasonable to assume that the nascent peptide would only further facilitate deviations from bulk behavior.

**Figure 1:**
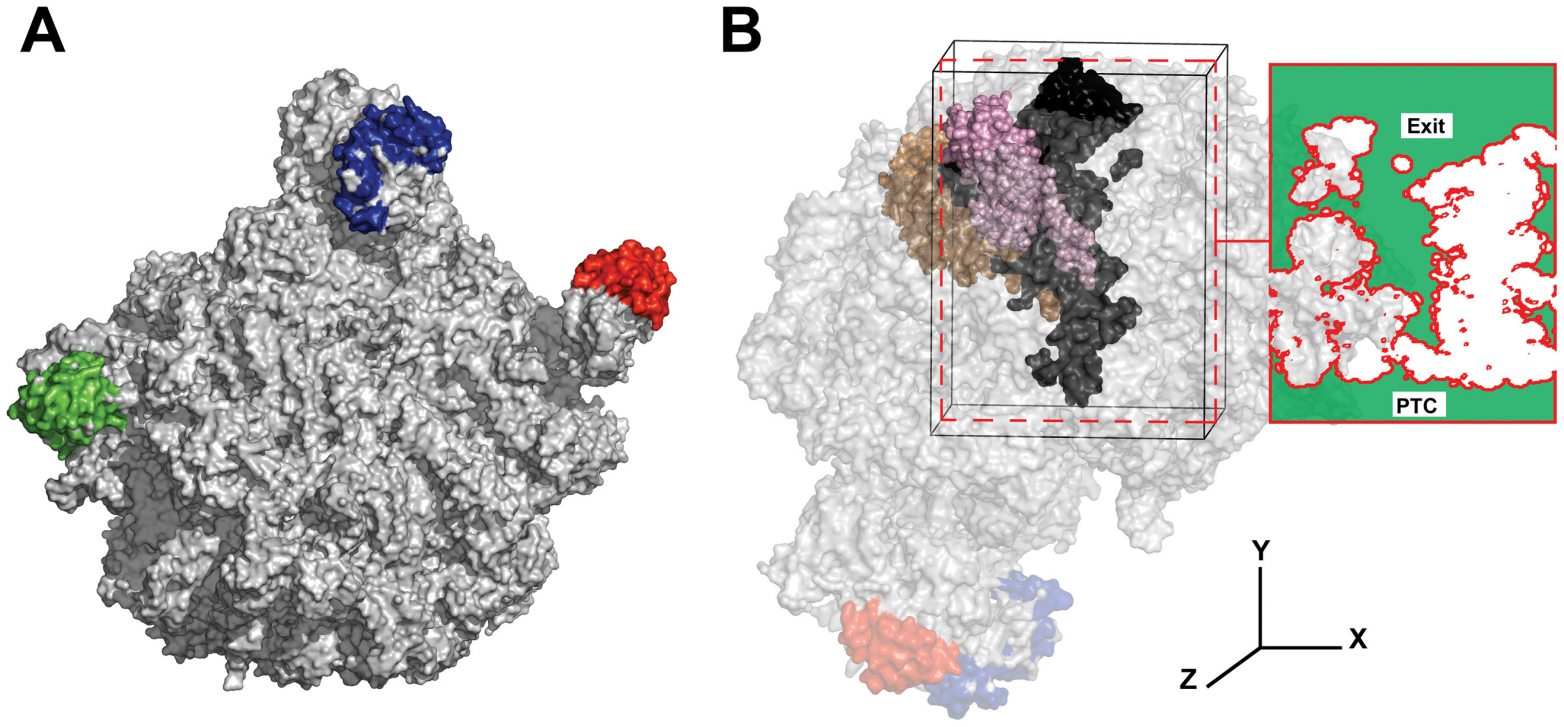
Explicit solvent simulation of the ribosome exit tunnel. The large ribosomal subunit (1S72) is shown in panel A from the standard “crown view” with the proteins L7ae, L5, and L11 shown in green, blue, and red respectively. Panel B shows the portion of the ribosome used in the molecular dynamics simulations (enclosed in the black box). The volume represented by the ribosome exit tunnel is shown in black with the exit at the top and the peptidyl transferase center at the bottom. The two proteins that protrude into the tunnel, L22 (pink) and L4 (brown), are shown as well. The dashed red box shows the plane of the tunnel between L22 and L4 (half way through the simulation box in the x dimension). The solid red box shows this plane with the area in the simulation box accessible to solvent colored green. All subsequent figures show this plane. The images in this figure were generated with MacPymol (DeLano, W.L. The PyMOL Molecular Graphics System (2002) DeLano Scientific, Palo Alto, CA, USA.).

In our model, 17,478 atoms were taken from the *Haloarcula marismortui* crystal structure [2] of the large subunit (as shown in figure 1 and discussed in the Methods section). Using atomistic molecular dynamics and large-scale distributed computing, we performed an aggregate of 40µs (10µs for our main system and each of 3 controls) of molecular dynamics simulation and then calculated a number of kinetic and thermodynamic properties of water inside the ribosome exit tunnel. We were able to observe bulk-like solvent properties beyond the mouth of the tunnel and significantly perturbed properties inside the tunnel. The results presented here are taken from a slice half way through the z-axis of the ribosome as shown in figure 1b. Although other slices show the same behavior, we chose this slice because it represents the plane between the portions of ribosomal proteins L4 and L22 that project into the tunnel (an area known as the constriction site).

**Figure 2:**
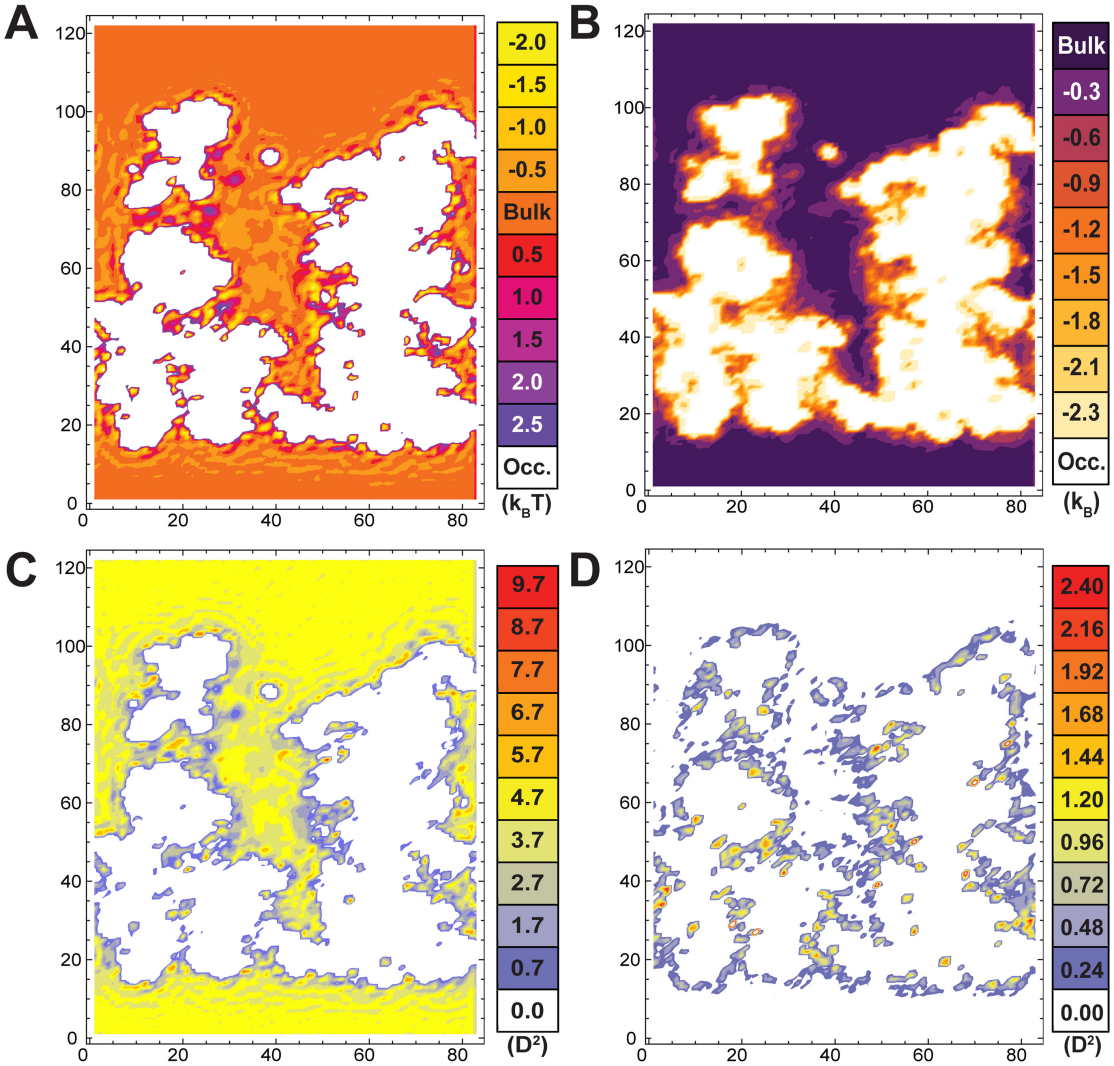
Thermodynamic analysis of solvent in the exit tunnel. This figure shows the thermodynamic properties of the solvent in the system. Panel A shows the potential of mean force for solvent. The x and y axes represent the position in the simulation box (in angstroms). The contours are spaced 0.5k_B_T apart. Panel B shows the solvent rotational entropy with contours 0.3k_B_ apart. Panel C shows the trace of the dipole fluctuation tensor while panel D shows the sum of the off diagonal elements of the dipole fluctuation tensor. Both panel C and D are in units of Debye squared.

A potential of mean force (PMF), or free energy of finding a water molecule at a specific location inside the exit tunnel was calculated (figure 2a). Such a PMF allows one to visualize spatial variations in solvation free energy relative to bulk, as they show the spatial dependence of the water density (on a logarithmic scale). Examination of this PMF reveals that solvent confined in the tunnel is perturbed relative to the bulk, with the most significant perturbations occurring within 5 angstroms of the tunnel surface. Within these regions, fluctuations of 3–4k_B_T are readily seen, while fluctuations of 0.5–1k_B_T are seen farther from the surface. These peaks imply the existence of subpopulations of ordered water that are not present in the bulk. Some of the regions experiencing these large fluctuations, such as the constriction site between the ribosomal proteins L4 and L22 are believed to be of importance to translation. In these regions, a heterogeneous water PMF could directly affect the solvation of nascent chains as well as modulate specific ribosome-protein or ribosome-small molecule interactions.

We also calculated the solvent rotational entropy as a function of position in the exit tunnel. Due to the importance of hydrogen bonding on solvent thermodynamics, rotational degrees of freedom are very important to water structure. The rotational entropy profile reveals that, like the PMF, the rotational degrees of freedom of solvent inside the exit tunnel are perturbed relative to bulk (figure 2b). Near the walls of the tunnel, we find significant reduction in rotational entropy relative to bulk: up to −2.1k_B_. This is indicative of ordered water that has access to only approximately 10% of the rotational space that is available to bulk. It should be noted that rotational entropy is a very sensitive measure of orientational structure. For example regions that differ from bulk by 0.3 k_B_ still suffer a 25% reduction in orientational freedom relative to bulk.

At a distance of approximately 5Å from the tunnel surface, the rotational entropy of solvent returns to the bulk value. This corresponds to the region of the tunnel that has an elevated solvation free energy relative to bulk. The absence of entropy contours in this area can be explained by consideration of our measure of rotational entropy. Regions with a more favorable solvation than bulk will not have more rotational freedom than bulk (the bulk value of rotational entropy should be a maximum for the system). When examining the relationship between rotational entropy and free energy we find that confinement to the ribosome exit tunnel facilitates a cooperative transition between highly ordered water with low internal energy and freely rotating water with high entropy (figure S1). This relationship allows us to use rotational entropy to define both bound and bulk-like states for water and examine where these two extrema occur in the ribosome exit tunnel. Considering water which samples 1 or 2 rotational states to be bound, and water than samples greater than 20 states to be bulk-like we can see (figure S1 panel B) that much of the water in the tunnel is between these two extremes of bound water and bulk-like continuum. This illustrates the inadequacy of the typical two-state water model to describe solvent behavior inside a nanoscopic cavity like the ribosome exit tunnel.

Calculation of rotational entropy requires one to partition rotational space. To ensure that our observations are robust with respect to this partitioning, an additional metric for rotational dynamics is useful. Since water has a permanent molecular dipole moment, solvent molecules with decreased orientational freedom should also have reduced dielectric susceptibility. Because of highly varying local electric fields (from the large amount of RNA and numerous counter ions in the system) as well as geometric heterogeneity of the tunnel, the calculation of an actual spatially varying dielectric constant by typical methods [44] is extremely difficult. Thus, we calculate the solvent dipole fluctuation tensor as a function of position in the tunnel as a proxy of this value. Comparing the elements of this tensor to the values found outside the tunnel suggests the degree to which dielectric constant would be perturbed.

Throughout the tunnel, the dipole moment fluctuation is significantly perturbed in a manner consistent with the PMF (plotted in figure 2c). We find significantly reduced dipole moment fluctuations near the tunnel surface, supporting the idea that solvent molecules localized to that region are ordered. We also find various “hot spots” with very high dipole fluctuation. These spots seem to correspond to locations where there is low rotational entropy but favorable free energy. This is indicative of an ordered water molecule that is rapidly switching between spatially disparate states (for example two orientations that may be 180 degrees apart). The off-diagonal elements of the tensor represent the degree to which polarization (reduced orientational freedom) in one dimension affects polarization in another direction, an effect that should only be non-zero in the case of significant solvent ordering. Indeed, we observe that the off diagonal elements of the tensor are equal to zero everywhere but near the edges of the tunnel (figure 2d), another illustration of significant solvent ordering that is consistent with the rotational entropy profile (figure 2b). Additionally, when comparing the individual components of the tensor, the dipole fluctuation is observed to be somewhat anisotropic (figure S5). Collectively, these results support our conclusion that highly perturbed rotational dynamics and dielectric behavior contribute to the complex solvation landscape inside the tunnel. These results imply that solvent confined to the ribosome does not behave as a continuous isotropic dielectric medium.

**Figure 3:**
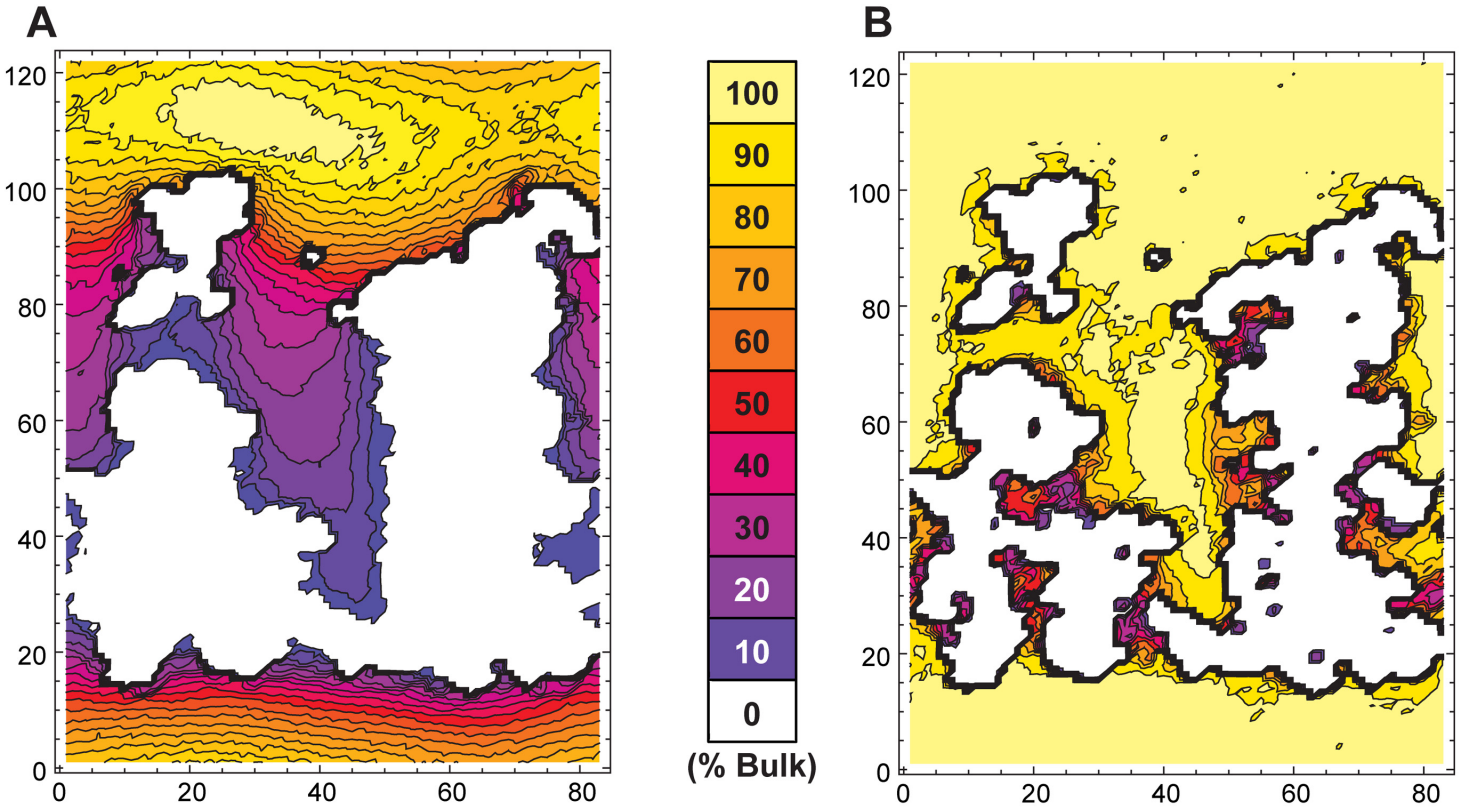
Kinetic analysis of solvent in the exit tunnel. Shown in panel A is the translational diffusion coefficient for water. Panel B shows the solvent rotational diffusion coefficient. The x and y axes represent the position in the simulation box (in angstroms). Contours are drawn in 10% intervals of the bulk value for both panels.

In addition to non bulk-like thermodynamics, we also observe that solvent confined in the ribosome exit tunnel exhibits significantly retarded kinetic properties as well. Figure 3a shows the translational diffusion coefficient of water in the tunnel. Within the tunnel, diffusion is greatly reduced. This is reasonable considering translational diffusion involves traversing the rugged solvation landscape shown in figure 2a. Furthermore, the diffusion coefficient was found to be highly anisotropic inside the tunnel, particularly along the tunnel's y-axis (figure S7). This result makes sense, as the tunnel surface is quite heterogeneous both in terms of geometry and surface chemistry. In addition to translational diffusion, we also calculate the rotational diffusion coefficient as a function of position in the tunnel (figure 3B). Rotational diffusion is marginally slowed throughout the tunnel, with severe restriction occurring only in specific locations. Indeed, there is a slight reduction in rotational diffusion around the edges of the tunnel with significant reduction only in regions with very low rotational entropy.

## Discussion

Our simulations predict that water confined to the ribosome exit tunnel is quite different from bulk solvent. The free energy of the water varies greatly with position in the tunnel relative to bulk, implying extensive water structure. Our data suggest that both the heterogeneity in the available rotational states as well as dielectric susceptibility make large contributions to the solvation landscape (although there may be other contributions to the solvation free energy, this hypothesis is consistent with all of our data). This rugged solvation landscape also serves to greatly restrict translational diffusion in the tunnel.

All of these data point to a different characterization of water than is commonly considered. On one hand, thinking of this water from a purely discrete structural perspective is not sufficient (although there are locations where water is essentially bound, this is not the majority of the confined solvent). On the other hand, this solvent is very different from bulk. It could perhaps be more accurately conceived as a slow-moving (retarded diffusion about thermal equilibrium) semi-structured fluid with dielectric properties somewhere between that of a macromolecule and bulk solvent. This water can even be considered a part of the structure of the ribosome, almost like an amorphous skin that affects macromolecular dynamics in cramped areas such as the exit tunnel.

### Experimental Implications

What might the effects of these properties be on biological processes? We find that our characterization of water is consistent with previous biochemical experiments investigating the behavior of nascent peptides in the tunnel. Our PMF shows that the upper region of the tunnel can be relatively well characterized as hydroscopic (shown as higher than bulk free energy of solvation throughout the middle of the tunnel) an observation consistent with the work of Lu et al [7]. Additionally, numerous studies show the intersection between L22 and L4 to be a place of importance for sequence dependent stalling [2], [4], [12], [14] as well as antibiotic binding [17], [18], [20], [21],[45]. Our data show that this region exhibits very complicated solvation behavior (as L4 and L22 present both polar and non-polar moieties to the tunnel). This solvation behavior could explain the predominance of this region as an important site for stalling and in particular, account for the large energy barrier tryptophan (conserved in SecM and tnaC stalling sequences) must cross in order to proceed through the tunnel during translation [12], [46].

Compaction of the nascent chain has been shown to exist in the region of the tunnel between the peptidyl-transferase center and the constriction site [10]–[12]. In the case of the SecM sequence this compaction is necessary for stalling. We believe that such compaction is quite compatible with our data. The surface of the tunnel in this region is largely hydrophilic and the SecM sequence is mostly hydrophobic in nature. The solvent confined between these two surfaces would have competing sets of thermodynamic demands on it, mediating a net repulsive force between the cavity and the peptide, thus favoring compaction. For hydrophilic sequences such as the tnaC stalling sequence, we would not predict for the chain to be collapsed or compacted. The low solvent entropy in this region of the exit tunnel implies that the difference in cost between solvent-solvent hydrogen bonds, and solvent-protein bonds is smaller than what would be observed in bulk. Thus a polar peptide such as tnaC would have less thermodynamic drive to form an alpha helix in this region of the tunnel. We would however expect for a polar sequence to become adsorbed onto the surface of the tunnel in this region. The solvent around the periphery of this section of the cavity is ordered (as evidenced by low rotational entropy and low dielectric shown in our data) and could only be easily displaced by polar residues of the nascent chain. This hypothesis is consistent with cryo-electron micrographs of tnaC in the exit tunnel, which show the tnaC peptide to be unfolded and making a number of tertiary interactions with the tunnel [14].

In addition to compaction of the nascent peptide in the region of the tunnel between the constriction site and the peptidyl transferase center, there have been recent observations of the nascent peptide folding into an alpha helix in the bottom portion of the tunnel close to the exit (a region known as the vestibule). Deutsch and coworkers have shown via chemical cross-linking studies that this region of the tunnel seems to promote the folding of a nascent peptide into an alpha helix [47], [48]. Additionally the hydrophilic (EAAAK)_5_ sequence inserted near the n-terminus of the peptide DPAP-B has been observed via cryo-electron microscopy to be in an alpha helical conformation in the vestibule [13]. Our results offer a potential mechanistic explanation for these new observations. We show that the vestibule of the ribosome exit tunnel has a favorable chemical potential for water relative to bulk. Forming a helix in this region would be thermodynamically favorable since maintaining an expanded conformation would involve displacing a larger number of favorably solvated waters, and forming a larger number of protein-water hydrogen bonds in a region where solvent-solvent hydrogen bonding is likely to be highly favored.

### Simulation Implications

How might this non-bulk-like water affect other attempts to study the ribosome using simulation techniques? The heterogeneous solvation landscape is likely to make prediction of binding free energies difficult, since desolvation would be a complicated function of position and orientation of the ligand in the exit tunnel (rather than a constant based on bulk measurements such as a partition coefficient). Thus, mean field approaches such as Poisson-Boltzmann and Generalized Born [49], which estimate the electrostatic solvation free energy based on a single isotropic dielectric constant, may have serious limitations due to the key role of correlations in solvent.

The ruggedness of the solvation landscape and slow kinetics are likely to make convergence difficult in free energy calculations using methods such as free energy perturbation or thermodynamic integration. This ruggedness would not only affect attempts to predict the affinity of small molecules to the ribosome exit tunnel (and thus confound attempts to discover new antibiotics) but also may play a role in examining the interaction of nascent peptides with the tunnel (such as the known stalling sequences). For this reason, it is expected to be difficult to obtain well-converged simulations of nascent peptides in tractable lengths of time. Systems in which there is extensive confinement (such as the ribosome exit tunnel) would greatly benefit from new advances in implicit solvation methodology, which could offer faster convergence and greater accuracy with respect to treatment of solvation.

We have discussed these results in the context of the ribosome, but one can also consider other implications. We have observed (please see text S1) that when water is confined to a “nonpolar ribosome” (one where we have zeroed the partial atomic charges and removed counterions) we also see highly perturbed thermodynamics, yet with different modes of organization in comparison to that seen in the original polar ribosome (figure S2). Thus, we have observed that restricted water entropy exists when water is confined to both polar and non-polar surfaces. Therefore, it is possible that our results can be generalized to a great number of important biological cavities. Among these are the chaperonin GroEL (when a substrate is encapsulated), the proteasome, the cavity created by the ribosomal chaperone trigger factor, and the translocon pore. For the ribosome, it should be noted that the degree of confinement the solvent experiences would increase as a nascent peptide traverses the exit tunnel (this would also hold true for trigger factor and the translocon pore). Considering the number of biological macromolecular complexes with some degree of confined water, we expect that the general principals described here may find wide applicability.

The great cost of extensive simulations such as those performed in this study makes a strong case for the use of simplified models to study large macromolecular complexes. Unfortunately our results show that the details neglected in current simplified models are likely to have large effects on protein thermodynamics and kinetics in nanoscopic cavities. The need for simplified models that are both computationally efficient and capture the physics of solvent on short length-scales is apparent. Some candidate models could include Kovelenko & Hirata's 3D-RISM methods as well as Koehl and Delarue's PBL method [50], [51]. Both attempt to treat the multi-body effects of solvent confined to nanoscopic cavities in an implicit manner. It still remains to be seen however if these promising models are computationally tractable for large-scale problems such as the study of the ribosome or chaperonins and similar macromolecular complexes.

## Methods

### Model Setup and Simulation Protocol

In order to model solvent dynamics in the ribosome exit tunnel, a cutout from the large ribosomal subunit (pdb code 1S72(2)) was constructed following Petrone et al [46]. The cutout was constructed by removing all atoms outside of a box (82.5Å by 85Å by 122Å) centered on the exit tunnel. This simulation box was constructed such that there was a 140 cubic nanometer bulk solvent region outside the mouth of the tunnel. The three missing residues (EVQ) from L39 were modeled in and equilibrated. The broken chains that arose from constructing the cutout were capped with dummy atoms. All crystallographic ions were included and 338 additional sodium atoms were added to balance the charge. The system was solvated with TIP3P water [52]. The system was then allowed to equilibrate for 1ns and more water molecules were added to achieve bulk density in the region outside the exit tunnel (the large amount of charge on the ribosome seemed to have an electrostrictive effect on the solvent). An additional 1ns of equilibration was then performed.

Five thousand 2ns molecular dynamics simulations were performed with the GROMACS simulation package [53] on the Folding@Home distributed computing network [54] using the Amber99phi force field [55]. These simulations were performed in the NVT ensemble with temperature controlled via the Berendsen thermostat at 298K [56] and a time step of 2 femtoseconds. Electrostatics were treated with the reaction field method [57] using a cutoff of 1 nm.

The above process was repeated four times. In the first set of simulations, the entire ribosome was held rigid (leaving only ions and water to move about freely). For the second set of simulations, the residues within 1nm of the exit tunnel were allowed to move while the rest of the ribosome atoms were held rigid. For the third set of simulations, the ribosome was allowed to maintain flexibility in the tunnel as before, but the simulation temperature was reduced to 100K (the crystallographic temperature). In the final set of simulations, all ribosome atoms were held rigid, but all ions were removed from the system and the charge on each ribosome atom was set to zero (effectively turning the ribosome into a porous hydrophobic surface). The results for the first set of simulations are presented in the main text while the results of the three additional control sets of simulations are presented in the supporting material (text S1, figure S2, figure S3, figure S6).

### Analysis

All thermodynamic and kinetic properties were calculated as a function of position within the simulation box by dividing the box into a three-dimensional grid and calculating a histogram based on water positions. The data was broken into 50 sets for block averaging (where the reported property is the average over all 50 blocks).

The two most obvious thermodynamic quantities of interest to water dynamics are the potential of mean force for water within the ribosome exit tunnel as well as the rotational entropy of water at different locations inside the tunnel. The potential of mean force using position within the tunnel as a reaction coordinate was calculated as shown in equation 1.
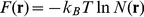
(1)where N(**r**) is the three dimensional histogram of water oxygen positions across 100 trajectories. The rotational entropy was calculated by binning each water molecule into one of 28 rotational states.
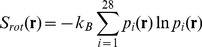
(2)After binning, the entropy was calculated from equation 2 where p_i_(**r**) is the probability of water at position **r** being in rotational state i. The rotational states were defined by partitioning space around the water oxygen into octants and assigning a state based on which octants contain the hydrogen atoms (thus 8 octants choose 2 hydrogen atoms gives 28 states).

Another important thermodynamic quantity is the local dielectric constant at different locations in the exit tunnel. Unfortunately, the traditional method for calculation of a local dielectric constant proposed by Kirkwood [44] would not apply in the non-isotropic environment of the ribosome exit tunnel. As a proxy of this value, the local dipole fluctuation tensor was calculated in a manner similar to that described in Lin et al. [58]. The elements of the tensor are given in equation 3.
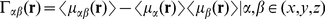
(3)Where <μ_αβ_(**r**)> is the average of the product of two components of the dipole moment of a water at position **r**, likewise <μ_α_(**r**)> is the average of a given component of the dipole moment of water at position **r**. In order to simplify this, yet not lose the generality of including the off-diagonal elements of the tensor we report the following quantities defined in equations 4 and 5:
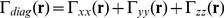
(4)

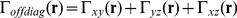
(5)We estimated the translational diffusion coefficient as a function of position in the tunnel by first assigning a water molecule to a grid position (as in the previous analysis) and measuring its mean square translational displacement 50 ps later. This was repeated for all trajectories and with the Einstein relation, yielded an average diffusion coefficient as a function of position in the exit tunnel. Additionally, we characterized rotational diffusion in a similar manner. For this method we measured the average cosine squared of the angle between the molecular dipole of water at time t and time t + 50ps.

Grid resolution of 1Å resolution was used for all calculations. Error analysis was performed by the bootstrap method (please see text S1 and figure S4).

## Supporting Information

Figure S1 Populations of water in the exit tunnel. Panel A of this figure shows the solvent rotational entropy plotted against the internal energy. The axes intersect at the location of the bulk solvent (calculated as the average in a 10 by 40 by 40 Angstrom slab beyond the mouth of the tunnel). The coloring corresponds to cutoffs for bound water (S_rot_ <0.7k_B_∼water that occupies only 1 or 2 rotational states) and bulk-like water (S_rot_ >3.0k_B_ {similar, tilde operator} water that occupies 20 or more rotational states). Panel B shows the solvent distribution inside the ribosome exit tunnel (see main text for description of the precise location) classified by this state description (red is bound, blue is bulk-like, and green is in between). Panel C shows a plot of rotational entropy versus the PMF (axes intersect at the location of bulk solvent). Blue corresponds to bulk-like solvent (within one standard deviation of the bulk value) while green corresponds to solvent with a free energy less favorable than bulk, and yellow corresponds to solvent with a free energy more favorable than bulk. The spatial distribution of these populations is shown in panel D for a slice half way through the tunnel.
(0.22 MB PNG)

Figure S2 Thermodynamic properties of a “non-polar ribosome” (charges and ions removed). Panel A shows the potential of mean force for solvent. The contours are spaces 0.5k_B_T apart. Panel B shows the solvent rotational entropy with contours 0.3k_B_ apart. Panel C shows the trace of the dipole fluctuation tensor while panel D shows the sum of the off diagonal elements of the dipole fluctuation tensor (in units of Debye squared).
(0.45 MB PNG)

Figure S3 The effect of allowing flexibility of the residues lining the ribosome exit tunnel. Panel A shows the solvent potential of mean force when the tunnel residues were allowed conformational flexibility. Contours are labeled at 0.5k_B_T intervals. Panel B shows the translational diffusion coefficient of the solvent with contours at drawn at 5% intervals of the bulk value. Panel C shows the PMF of the same system with the temperature reduced to 100K (the crystallographic temperature) and panel D shows the translational diffusion coefficient (contours drawn at 0.05% intervals from the bulk value in panel B).
(0.60 MB PNG)

Figure S4 Error analysis for thermodynamic data. Here we show the statistical error computed from 100 bootstrap samples of 50 trajectories. Panel A shows the error in the PMF as a function of PMF (relative to bulk), while panel B shows the error in the rotational entropy as a function of rotational entropy. The vertical line indicates the location of the bulk value. Panel C shows the spatial distribution of error in the PMF with contours drawn at 0.01k_B_T intervals.
(0.21 MB PNG)

Figure S5 Dipole fluctuation tensor for standard simulations. This figure shows the components of the dipole fluctuation tensor (calculated as described in materials and methods). Contours are drawn at 0.1 Debye squared. Only six tensor elements are shown, as it is symmetric by construction.
(0.19 MB PNG)

Figure S6 Dipole fluctuation tensor for non-polar ribosome. This figure shows the components of the dipole fluctuation tensor for the “non-polar” ribosome calculated by the method described in the main text. Contours are drawn at 0.1 Debye squared. Only six tensor elements are shown, as it is symmetric by construction.
(0.20 MB PNG)

Figure S7 Components of solvent diffusion. Shown here is the solvent translational diffusion from main text figure 3A separated into individual dimensions. Contours are drawn in 10% intervals of the maximum value (which occurs in the bulk). This serves to demonstrate that the diffusion is highly anisotropic in the ribosome exit tunnel.
(0.41 MB PNG)

Text S1 Included here are the results and discussion of control simulations, error analysis, and a supplemental analysis of the PMF and rotational entropy, showing how they can be used to classify sub-populations of solvent.
(0.03 MB DOC)
