# Supplementary figures and images for: Non-Bulk-Like Solvent Behavior in the Ribosome Exit Tunnel
Source: PLoS Comput Biol. 2010 Oct 21;6(10):e1000963. doi: 10.1371/journal.pcbi.1000963 (PMC2958802; doi:10.1371/journal.pcbi.1000963)

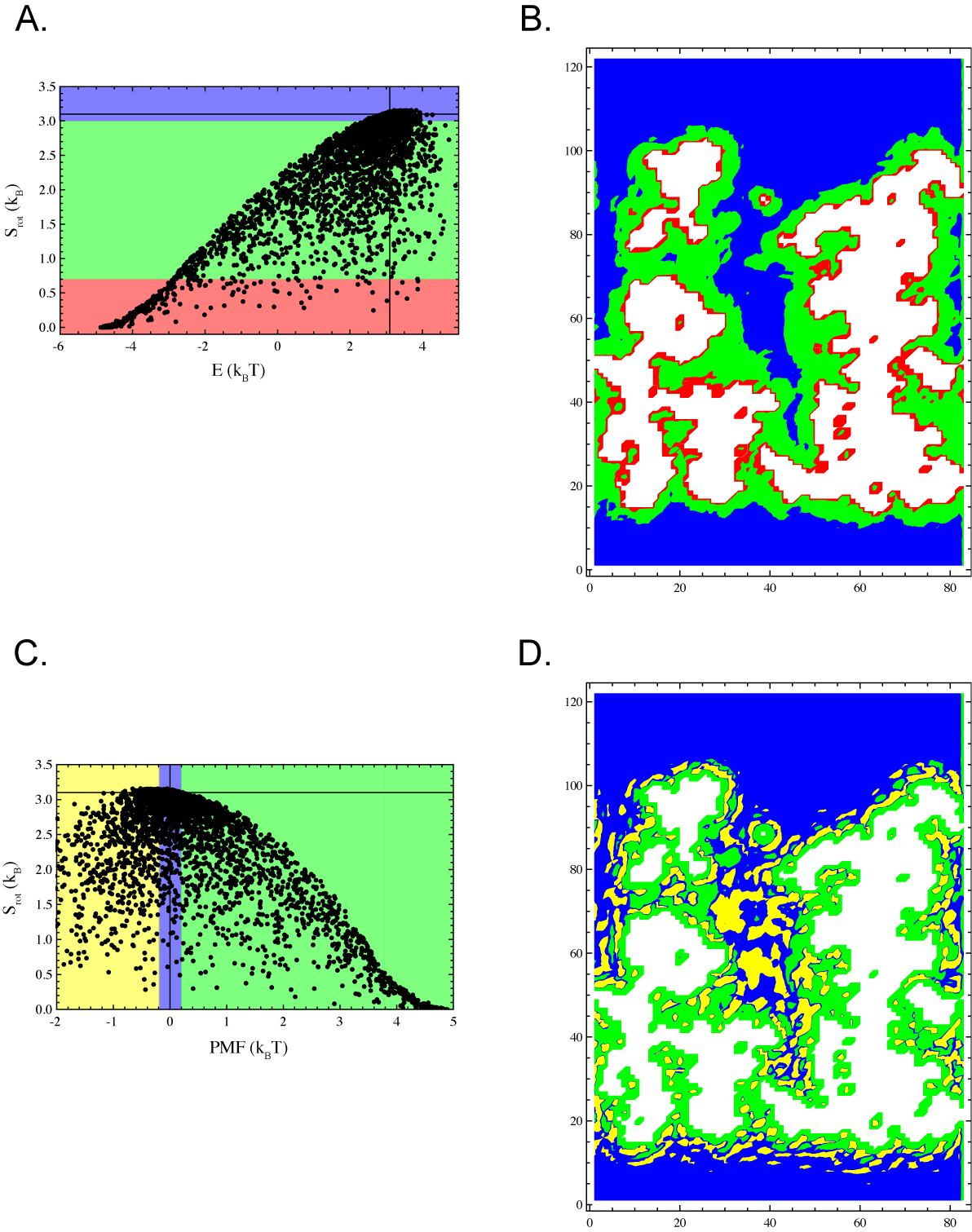

Supplement: Figure S1 — Populations of water in the exit tunnel. Panel A of this figure shows the solvent rotational entropy plotted against the internal energy. The axes intersect at the location of the bulk solvent (calculated as the average in a 10 by 40 by 40 Angstrom slab beyond the mouth of the tunnel). The coloring corresponds to cutoffs for bound water (Srot <0.7kB∼water that occupies only 1 or 2 rotational states) and bulk-like water (Srot >3.0kB {similar, tilde operator} water that occupies 20 or more rotational states). Panel B shows the solvent distribution inside the ribosome exit tunnel (see main text for description of the precise location) classified by this state description (red is bound, blue is bulk-like, and green is in between). Panel C shows a plot of rotational entropy versus the PMF (axes intersect at the location of bulk solvent). Blue corresponds to bulk-like solvent (within one standard deviation of the bulk value) while green corresponds to solvent with a free energy less favorable than bulk, and yellow corresponds to solvent with a free energy more favorable than bulk. The spatial distribution of these populations is shown in panel D for a slice half way through the tunnel. (0.22 MB PNG) [file pcbi.1000963.s001.png]

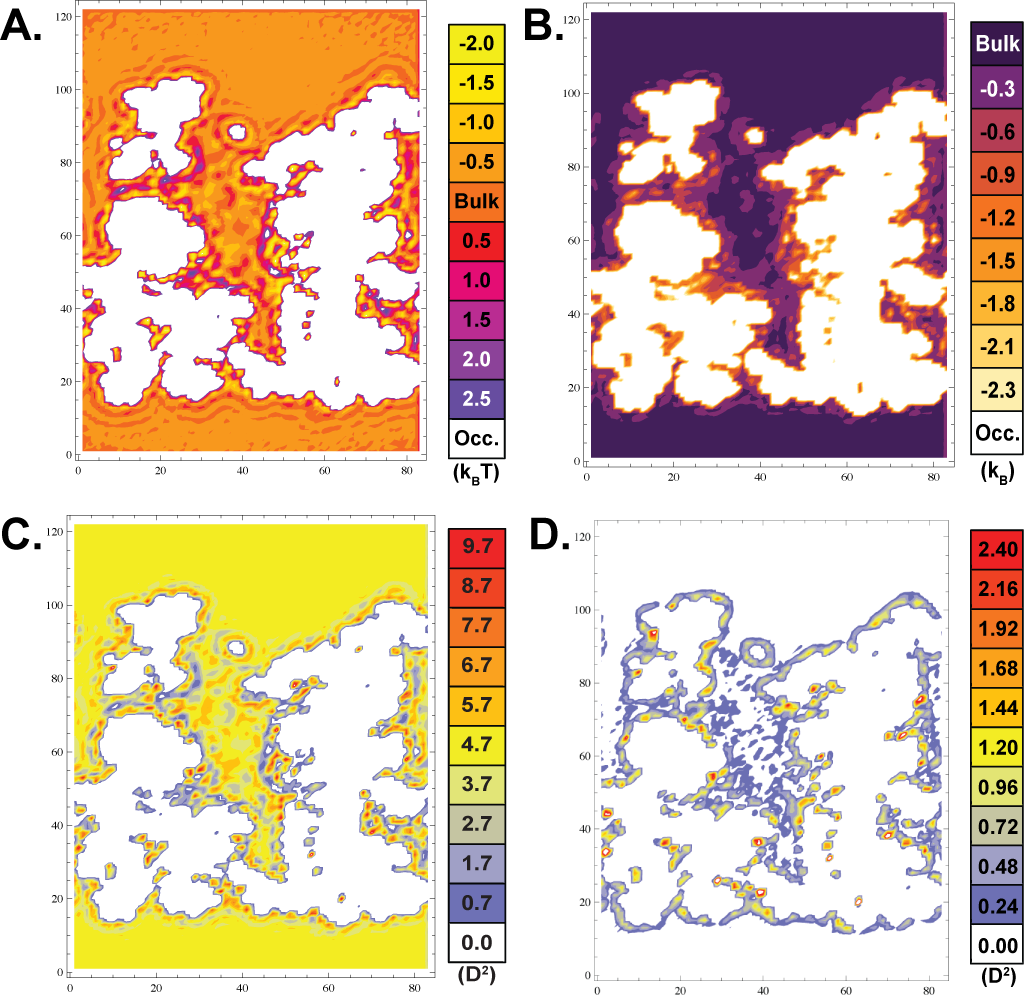

Supplement: Figure S2 — Thermodynamic properties of a “non-polar ribosome” (charges and ions removed). Panel A shows the potential of mean force for solvent. The contours are spaces 0.5kBT apart. Panel B shows the solvent rotational entropy with contours 0.3kB apart. Panel C shows the trace of the dipole fluctuation tensor while panel D shows the sum of the off diagonal elements of the dipole fluctuation tensor (in units of Debye squared). (0.45 MB PNG) [file pcbi.1000963.s002.png]

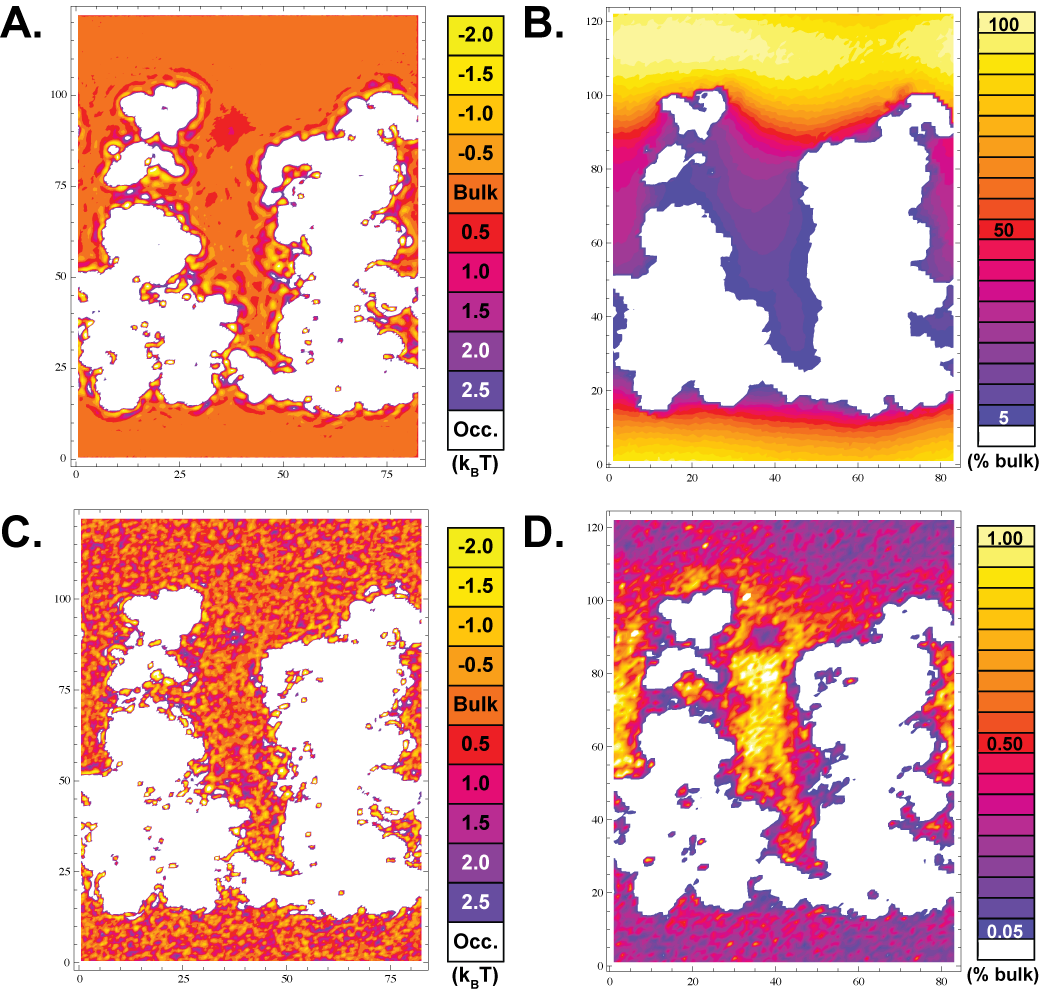

Supplement: Figure S3 — The effect of allowing flexibility of the residues lining the ribosome exit tunnel. Panel A shows the solvent potential of mean force when the tunnel residues were allowed conformational flexibility. Contours are labeled at 0.5kBT intervals. Panel B shows the translational diffusion coefficient of the solvent with contours at drawn at 5% intervals of the bulk value. Panel C shows the PMF of the same system with the temperature reduced to 100K (the crystallographic temperature) and panel D shows the translational diffusion coefficient (contours drawn at 0.05% intervals from the bulk value in panel B). (0.60 MB PNG) [file pcbi.1000963.s003.png]

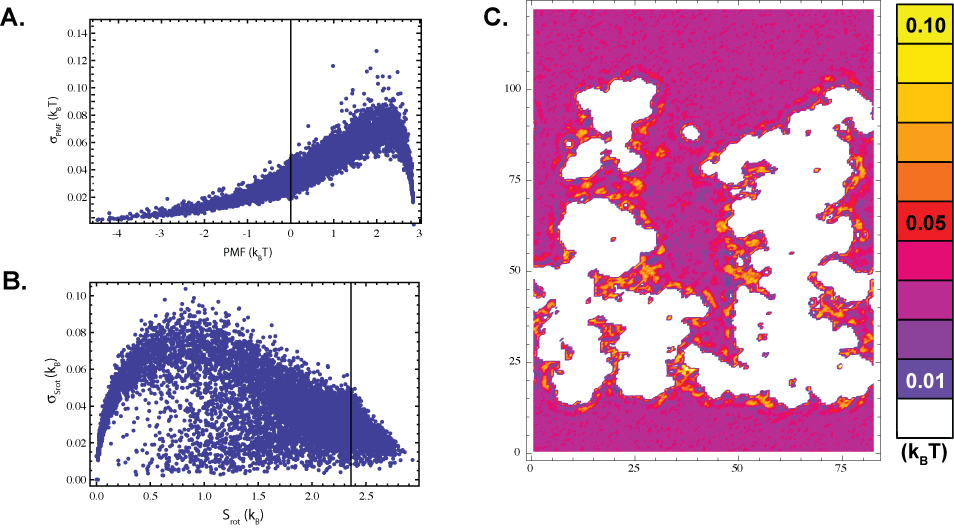

Supplement: Figure S4 — Error analysis for thermodynamic data. Here we show the statistical error computed from 100 bootstrap samples of 50 trajectories. Panel A shows the error in the PMF as a function of PMF (relative to bulk), while panel B shows the error in the rotational entropy as a function of rotational entropy. The vertical line indicates the location of the bulk value. Panel C shows the spatial distribution of error in the PMF with contours drawn at 0.01kBT intervals. (0.21 MB PNG) [file pcbi.1000963.s004.png]

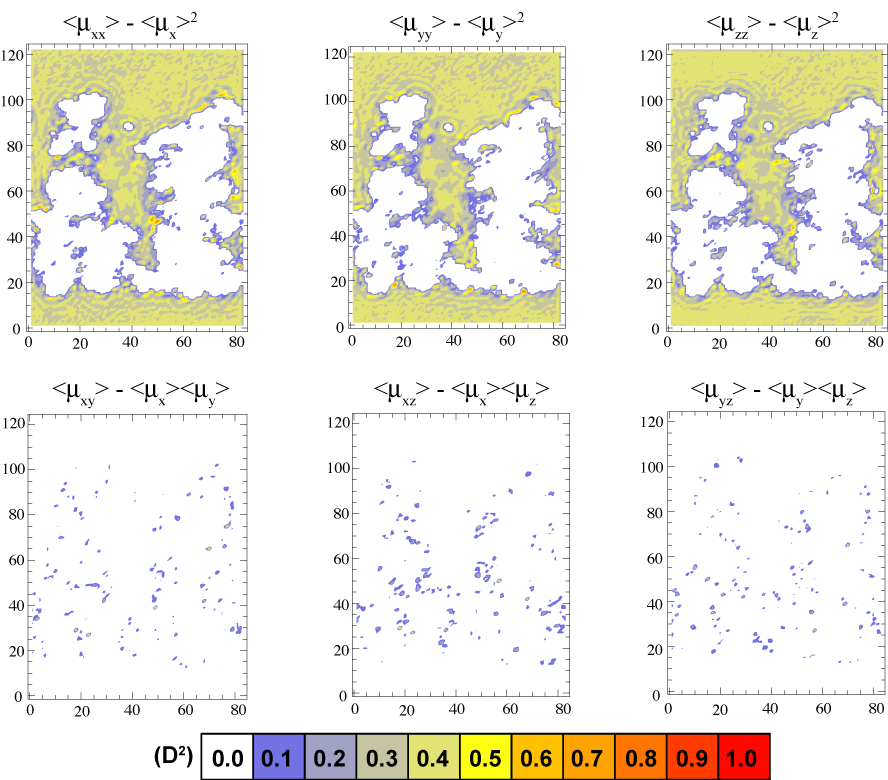

Supplement: Figure S5 — Dipole fluctuation tensor for standard simulations. This figure shows the components of the dipole fluctuation tensor (calculated as described in materials and methods). Contours are drawn at 0.1 Debye squared. Only six tensor elements are shown, as it is symmetric by construction. (0.19 MB PNG) [file pcbi.1000963.s005.png]

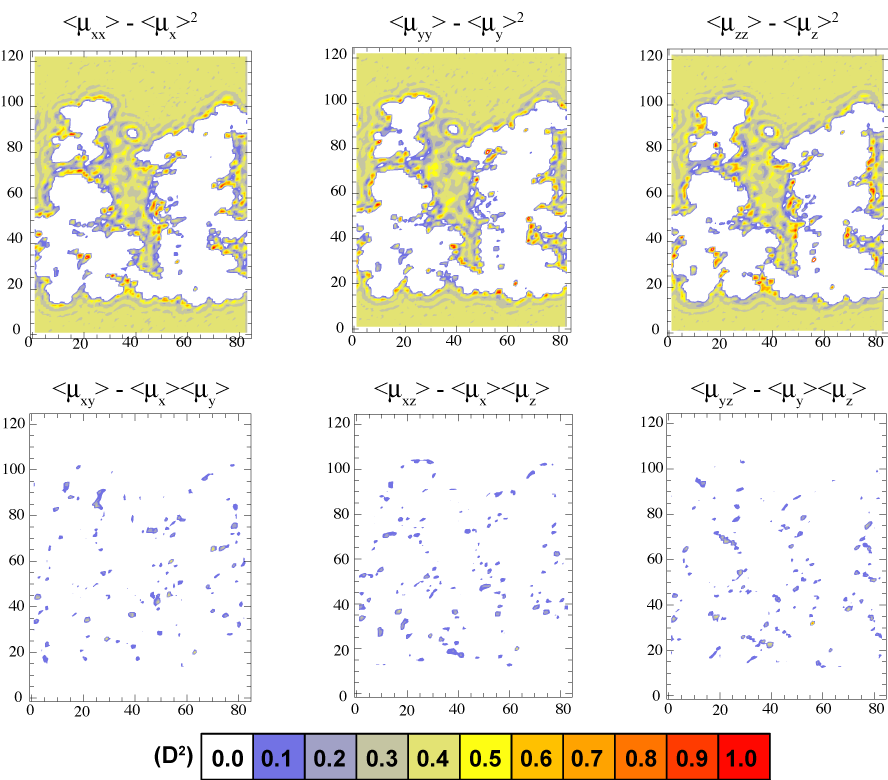

Supplement: Figure S6 — Dipole fluctuation tensor for non-polar ribosome. This figure shows the components of the dipole fluctuation tensor for the “non-polar” ribosome calculated by the method described in the main text. Contours are drawn at 0.1 Debye squared. Only six tensor elements are shown, as it is symmetric by construction. (0.20 MB PNG) [file pcbi.1000963.s006.png]

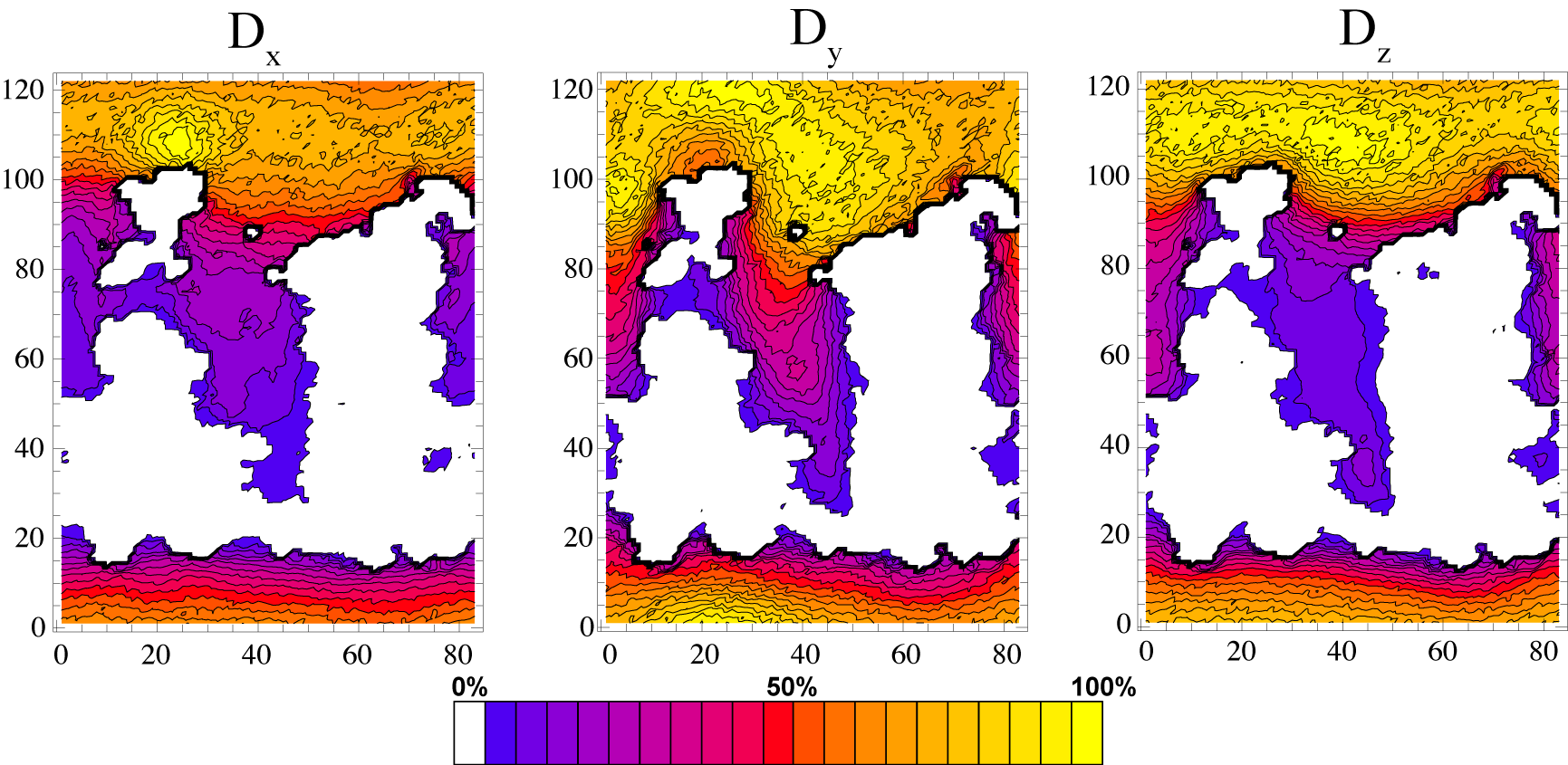

Supplement: Figure S7 — Components of solvent diffusion. Shown here is the solvent translational diffusion from main text figure 3A separated into individual dimensions. Contours are drawn in 10% intervals of the maximum value (which occurs in the bulk). This serves to demonstrate that the diffusion is highly anisotropic in the ribosome exit tunnel. (0.41 MB PNG) [file pcbi.1000963.s007.png]
