# Supplementary material for: Non-Bulk-Like Solvent Behavior in the Ribosome Exit Tunnel
Source: PLoS Comput Biol. 2010 Oct 21;6(10):e1000963. doi: 10.1371/journal.pcbi.1000963 (PMC2958802; doi:10.1371/journal.pcbi.1000963)
Supplement: Text S1 — Included here are the results and discussion of control simulations, error analysis, and a supplemental analysis of the PMF and rotational entropy, showing how they can be used to classify sub-populations of solvent. (0.03 MB DOC) [file pcbi.1000963.s008.doc]

Supplementary Material for “Non-bulk-like solvent behavior in the ribosome exit tunnel”

Del Lucent*, Christopher Snow*, Colin Echeverría Aitken*, Vijay S. Pande*^†‡§^

*Biophysics Program, ^†^Department of Chemistry, and ^‡^Department of Structural Biology, Stanford University, Stanford CA 94305

**^§^Corresponding Author:**

Prof. Vijay S. Pande

Department of Chemistry, Department of Structural Biology

Stanford University, Stanford CA 94305

Phone: 650-723-3660

Fax: 650-725-0259

URL: http://pande.stanford.edu

E-mail: pande@stanford.edu

# Rotational Entropy: Supplementary Analysis

We observed that the free energy of solvation and the rotational entropy (S_rot_) of the solvent confined to the ribosome exit tunnel are significantly perturbed relative to bulk. To further examine this relationship we have plotted the rotational entropy against the internal energy (calculated from the definition of Helmholtz free energy ). Although there is considerable scatter in the data, we can see that rotational entropy seems to show the expected sigmoidal relationship between bound solvent (low S_rot_) and bulk-like solvent (high S_rot_) (figure S1a). We also see that there is a tradeoff between energetically favorable low entropy states and energetically unfavorable high entropy states. We can use rotational entropy to classify the solvent in the system. If we define water only occupying 1 or 2 rotational states as bound (red area) and water within 1 standard deviation of the average value of S_rot_ outside of the tunnel as bulk (20 or more rotational states), we can visualize where our confined water differs from these two standard characterizations of solvent. We can see from the spatial distribution of these points (figure S1b) that the majority of the solvent in the ribosome exit tunnel is intermediate between bulk-like water and tightly bound water.

We can also classify these populations of solvent using our PMF (figure S1c,d). Values within one standard deviation of the average free energy beyond the mouth of the tunnel are considered bulk-like (colored blue), values greater than bulk are colored green and values less than bulk are colored yellow. With this classification scheme we can see that in addition to a number of favorable hydration sites (whose waters are mostly unbound by our definition based on rotational entropy) the overall hydrophilic character of the tunnel can be visualized by the copious areas inside the middle of the tunnel with greater chemical potential for solvent than bulk.

# Control Simulations: Non-polar Tunnel

In the main manuscript, we visualize the solvation landscape of water in the ribosome exit tunnel via the PMF shown in figure 2a. Because the ribosome exit tunnel is mostly lined with RNA, it can be considered a polar surface. In order to determine the extent that polarity is responsible for the water PMF, we performed a simulation wherein all ions were removed and all ribosome charges were set to zero. The resulting solvent PMF is shown in figure S2a. This PMF is similar to that shown in figure 2a in the main text in that water structure is perturbed relative to bulk (even more so than in the case of a regular ribosome with non zero charge). In addition, the location and depth of many solvation shells have shifted. This seems to be a reasonable result considering that water lining a polar surface and water lining a non-polar surface exhibits two different modes of organization (see main text references 32-35). The water is still ordered, but now the ordering arises in part from the solvent’s inability to hydrogen bond with the tunnel surface. This solvent is highly structured because the number of hydrogen bonding partners (and thus accessible rotational states) is diminished. This is supported by examining the rotational entropy and dielectric properties of this system (figure S2b,c). The rotational entropy is highly perturbed relative to bulk. We can see an extended low entropy region outlining much of the surface. This contributes to an unfavorable free energy of solvation near the non-polar surface (since there are now no hydrogen bonding partners near the surface). The dipole moment fluctuation data also support these observations, as regions of favorable free energy also have large fluctuations in dipole moment whereas regions of unfavorable free energy also have low fluctuations (figure S2c). In addition, the dipole moment fluctuation tensor is highly anisotropic in some areas (figure S6). Finally, the extensive solvent ordering that results from solvent receding from the non-polar ribosome is supported by the non-zero values of the off diagonal elements of the dipole fluctuation tensor. These results imply that although the mode of organization is different, solvent confined to a non-polar cavity is also quite different from bulk.

# Control Simulations: Flexible Tunnel

Although the results shown in the main text are compelling, one must ask do these properties persist if the residues lining the tunnel are allowed conformational flexibility. Upon repeating the simulation with a flexible tunnel, a qualitatively similar PMF is still observed in which most of the solvent confined to the tunnel is perturbed relative to bulk (figure S3a). Quantitatively, some of the fine details are washed out and previously occluded regions (such as helix 24 of the 23S rRNA near the exit of the tunnel) now merely appear as regions of less favorable solvation. Nonetheless, we see that in spite of having conformational flexibility, the water remains structured relative to bulk. In addition, we see the same general trend of reduced translational diffusion throughout tunnel (figure S3c).

It should be noted that when these simulations are performed at 100K (the crystallographic temperature) the PMF inside and outside of the tunnel are essentially the same (and varies sharply on very small length scales, figure S3b). Although the simple TIP3P water model is not parameterized to yield proper thermodynamic properties at such depressed temperature, one can still envision qualitatively that water in the tunnel seems to look similar to bulk water at a lower temperature. At this low temperature, diffusion is reduced by two orders of magnitude. Although there appears to be a marginal difference in diffusion coefficient between the inside and outside of the tunnel (figure S3d) at such a depressed temperature this difference is not likely to be significant.

# Error Analysis

Statistical error was quantified by dividing the 5000 molecular dynamics trajectories into 50 blocks and re-sampling these 50 blocks using a bootstrap with 100 iterations. The reported error is the standard deviation of the distribution from the bootstrap re-sampling. Average error in the PMF is 0.03k_B_T in the bulk region. The error increases with increasing free energy up to 0.15k_B_T and then drops off (figure S4a). This drop is attributed to regions that are nearly always devoid of solvent. The average error in the rotational entropy is 0.02k_B_ in the bulk region. The maximum error is about 0.1k_B_ (figure S4b). The regions of largest error are near the surface of the tunnel, which is to be expected, as this is where the most significant deviations from bulk behavior occur (figure S4c). None-the-less, for both the PMF and the rotational entropy, the upper bound on the error is still quite low.
